# CSF and Serum Levels of Inflammatory Markers in PD: Sparse Correlation, Sex Differences and Association With Neurodegenerative Biomarkers

**DOI:** 10.3389/fneur.2022.834580

**Published:** 2022-02-25

**Authors:** Stefanie Lerche, Milan Zimmermann, Isabel Wurster, Benjamin Roeben, Franca Laura Fries, Christian Deuschle, Katharina Waniek, Ingolf Lachmann, Thomas Gasser, Meike Jakobi, Thomas O. Joos, Nicole Schneiderhan-Marra, Kathrin Brockmann

**Affiliations:** ^1^Department of Neurodegeneration, Center of Neurology, Hertie-Institute for Clinical Brain Research, University of Tuebingen, Tuebingen, Germany; ^2^German Center for Neurodegenerative Diseases, University of Tuebingen, Tuebingen, Germany; ^3^Roboscreen GmbH, Leipzig, Germany; ^4^Natural and Medical Sciences Institute at the University of Tübingen (NMI), Reutlingen, Germany

**Keywords:** cytokines, CSF, serum, tau, alpha-synuclein, NFL

## Abstract

**Background:**

An involvement of the central-nervous and peripheral, innate and adaptive immune system in the pathogenesis of Parkinson's disease (PD) is nowadays well established.

**Objectives:**

We face several open questions in preparation of clinical trials aiming at disease-modification by targeting the immune system: Do peripheral (blood) inflammatory profiles reflect central (CSF) inflammatory processes? Are blood/CSF inflammatory markers associated with CSF levels of neurodegenerative/PD-specific biomarkers?

**Methods:**

Using a multiplex assay we assessed 41 inflammatory markers in CSF/serum pairs in 453 sporadic PD patients. We analyzed CSF/serum correlation as well as associations of inflammatory markers with clinical outcome measures (UPDRS-III, H&Y, MoCA) and with CSF levels of α-synuclein, Aβ_1−42_, *t-*Tau, p181-Tau and NFL. All analyses were stratified by sex as the immune system shows relevant sex-specific differences.

**Results:**

Correlations between CSF and serum were sparse and detected in only 25% (9 out of 36) of the analysable inflammatory markers in male PD patients and in only 38% (12 out of 32) of female PD patients. The most important pro-inflammatory mediators associated with motor and cognitive decline as well as with neurodegenerative/PD-specific biomarkers were FABP, ICAM-1, IL-8, MCP-1, MIP-1-beta, and SCF. Results were more robust for CSF than for serum.

**Interpretation:**

Levels of central-nervous and peripheral inflammatory markers might be regulated independently of each other with CSF inflammatory markers reflecting CNS pathology more accurately than peripheral markers. These findings along with sex-specific characteristics have to be considered when designing clinical trials aiming at disease-modification by targeting the immune system.

## Introduction

Parkinson's disease (PD) is a multifactorial disorder with age, genetics, environmental and life-style factors contributing to disease manifestation and clinical trajectories. Moreover, a growing number of epidemiological and genetic studies as well as post-mortem and fluid-biomarker analyses provide evidence for a relevant influence of inflammation on incidence and progression in PD (1–4). An involvement of the central-nervous and peripheral, innate and adaptive immune system has been detected (5). The activation of microglia as representative of the cerebral innate immune system was shown post-mortem as well as *in-vivo* in PD patients by Positron Emission Tomography studies (TSPO-PET, 18F-FEPPA-PET) and by increased levels of cytokines in CSF (2, 6). Microglia can be activated by Damage-Associated Molecular Patterns (DAMPs), which are generated by damaged cells, misfolded proteins, and protein aggregates. In PD, α-synuclein acts as DAMP resulting in microglia activation with induction of a neuroinflammatory response and release of cytokines/chemokines (7). Moreover, there is increasing evidence for the involvement of the peripheral innate and adaptive immune system in the pathophysiology of PD. It was shown that α-synuclein induces inflammasome-related cytokine production in the periphery and specific α-synuclein peptides act as antigenic epitopes resulting in helper and cytotoxic T cell responses in PBMCs from patients with PD (8, 9).

Several post-mortem and biofluid (blood, CSF) studies reported increased inflammatory profiles to be associated with clinical subtypes of PD, promoting an accelerated motor and non-motor phenotype (3, 10–13). Recent evidence highlights that the involvement of inflammation in PD is maximized in the early disease stages and maintains a chronic profile during the course of the disease (14, 15).

Despite this clear role for inflammation in the pathogenesis of PD, we face several open questions in preparation of clinical trials aiming at disease-modification by targeting the immune system: 1. Do peripheral (blood) inflammatory profiles reflect central (CSF) inflammatory processes indicative of a cross-talk between periphery and brain? 2. Are blood/CSF inflammatory markers associated with CSF levels of neurodegenerative/PD-specific biomarkers such as α-synuclein, Aβ_1−42_, Tau and NFL? Knowledge on these questions will help to stratify patients and enrich cohorts for clinical trials.

## Methods

### Participants

Between 2005 and 2018, CSF/serum pairs of 453 sporadic PD patients recruited from the outpatient clinic and/or ward for PD at the University Hospital of Tübingen were collected. Fourty-eight neurodegenerative healthy elderly (spouses, volunteers) served as control individuals.

Male PD patients presented with mean age of 66 years, mean age at onset of 59 years, mean disease duration of 7 years, mean HY of 2.2, mean UPDRS-III of 28, mean MoCA of 25, mean LEDD of 619. Mean CSF levels in pg/ml were as follows: Aβ_1−42_ 676, *t-*Tau 230, p-Tau 41, NFL 1025, total α-synuclein 569. Female PD patients presented with mean age of 66 years, mean age at onset of 59 years, mean disease duration of 7 years, mean HY of 2.1, mean UPDRS-III of 26, mean MoCA of 25, mean LEDD of 533. Mean CSF levels in pg/ml were as follows: Aβ_1−42_ 684, *t-*Tau 257, p-Tau 43, NFL 1143, total α-synuclein 689. For more demographic and clinical details (see Supplementary Table 1).

### Clinical Investigations

All participants were examined by a neurologist specialized in movement disorders. Diagnosis of PD was defined according to UK Brain Bank Society Criteria (16, 17). Disease duration was defined as interval between onset of PD and biosample collection. PD patients were assessed in the dopaminergic ON state. We assessed severity of motor symptoms using part III of the Unified Parkinson's disease Rating Scale (UPDRS-III), from 2006 to 2008 the old version and from 2009 on the MDS-UPDRS (18). Disease stage was categorized by the modified Hoehn and Yahr Scale (H&Y) (19). Cognitive function was tested using the Montreal Cognitive Assessment (MoCA) (20) and/or the Mini Mental Status Examination (MMSE) (21). Since the MoCA was available only from 2009 on, all previously obtained MMSE scores were converted into MoCA equivalent scores according to a published algorithm (22).

### Collection of CSF and Serum Samples

Spinal tap for CSF collection and venous blood sampling were performed directly one after another between 9.00 AM and 1.00 PM. Samples were taken from the bedside and centrifuged within 60 min and frozen at −80°C within 90 min after collection. Until 2013, we used polypropylene tubes from Sarstedt (Article Nr. 72.730.406) and from 2013 on, we used low protein-binding polypropylene cryovial 2D barcode cryovials FluidX (Article Nr. 68-0703-01) for storage. Samples with abnormal routine CSF diagnostics (erythrocytes >1/μl, white blood cell count >5 cells/μl, immunoglobulin subtype G index >0.7) were excluded.

### CSF and Serum Measurement of Inflammatory Markers

Levels of 41 inflammation-associated markers were measured in CSF/serum pairs using the multiplexed immunoassay by Myriad RBM, Austin, TX, USA (http://rbm.myriad.com). Mean storage-time until measurement was 6 years. Storage-time showed a negative correlation in CSF for ICAM-1 (−0.155 *p* = 0.004), Interleukin-4 (−0.146 *p* = 0.007), Interleukin-7 (−0.255 *p* < 0.001), PSA-f (−0.155 *p* = 0.029) and TF (−0.108 *p* = 0.045) and a negative correlation in serum for CKMB (−0.115 *p* = 0.032) and BDNF (−0.214 *p* < 0.001). For measurements, samples were thawed at room temperature, vortexed, spun at 18.000 x g for 1 min and pipetted into a master microtiter plate. After dilution with assay diluents in a manner of 1:5, an aliquot of 10 μl diluted sample was introduced into one of the capture microsphere multiplexes followed by incubation at room temperature for 1 h. Reporter antibodies were added followed by incubation for an additional hour at room temperature. Streptavidin-phycoerythrin solution was added followed by incubation for another hour at room temperature. For control purposes, calibrators and controls were included on each microtiter plate. Standard curve, control, and sample QC were performed to ensure proper assay performance. Samples were tested in singles. Analysis was performed using the Luminex 100/200 instrument. Data were interpreted using the software developed and provided by Myriad RBM.

The Lower Limit of Quantitation (LLOQ) means the lowest concentration of an analyte in a sample that can be reliably detected and at which the total error meets the laboratory's requirements for accuracy. NMI's requirement for accuracy is the concentration of an analyte at which the coefficient of variation of replicate standard samples is 30%.

CSF LLOQ: CA 125 U/ml: 2; IgE U/ml: 2,04; TSH ulU/ml: 0,0195; Alpha Fetoprotein pg/ml: 110; CEA pg/ml: 95,8; CKMB pg/ml: 157; FABP pg/ml: 618; Factor VII pg/ml: 562; GH pg/ml: 36,2; ICAM-1 pg/ml: 644; Leptin pg/ml: 60,4; MMP-3 pg/ml: 42,4; MMP-9 pg/ml: 7,840; PSA-f pg/ml: 4,32; TF pg/ml: 89,6; TNF-alpha pg/ml: 3,58; Brain-Der Neu Fac pg/ml: 23,2; ENA 78 pg/ml: 19,2; GM-CSF pg/ml: 19,1; IL-1alpha pg/ml: 1; IL-1beta pg/ml: 0,358; IL-2 pg/ml: 6; IL-3 pg/ml: 4,08; IL-4 pg/ml: 5,22; IL-5 pg/ml: 2,3; IL-6 pg/ml: 0,862; IL-7 pg/ml: 8,44; IL-8 pg/ml: 2,74; IL-10 pg/ml: 2,28; IL-12 p40 pg/ml: 73,8; IL-12 p70 pg/ml: 18,1; IL-13 pg/ml: 2,04; IL-15 pg/ml: 187; IL-16 pg/ml: 12,3; IL-18 pg/ml: 12,4; Lymphotactin pg/ml: 85,4; MCP-1 pg/ml: 8,66; MDC pg/ml: 5,82; MIP-1 beta pg/ml: 13,6; SCF pg/ml: 26,2; TNF-beta pg/ml: 2,62; TPO pg/ml: 420.

Serum LLOQ: CA 125 U/ml: 5; IgE U/ml: 5,1; TSH ulU/ml: 0,0489; Alpha Fetoprotein pg/ml: 276; CEA pg/ml: 240; CKMB pg/ml: 394; FABP pg/ml: 1,550; Factor VII pg/ml: 1,410; GH pg/ml: 90,5; ICAM-1 pg/ml: 1,610; Leptin pg/ml: 151; MMP-3 pg/ml: 106; MMP-9 pg/ml: 19,600; PSA-f pg/ml: 10,8; TF pg/ml: 224; TNF-alpha pg/ml: 8,95; Brain-Der Neu Fac pg/ml: 58; ENA 78 pg/ml: 47,9; GM-CSF pg/ml: 47,7; IL-1alpha pg/ml: 2,5; IL-1beta pg/ml: 0,895; IL-2 pg/ml: 15; IL-3 pg/ml: 10,2; IL-4 pg/ml: 13,1; IL-5 pg/ml: 5,75; IL-6 pg/ml: 2,16; IL-7 pg/ml: 21,1; IL-8 pg/ml: 6,85; IL-10 pg/ml: 5,7; IL-12 p40 pg/ml: 185; IL-12 p70 pg/ml: 45,2; IL-13 pg/ml: 5,1; IL-15 pg/ml: 468; IL-16 pg/ml: 30,7; IL-18 pg/ml: 31; Lymphotactin pg/ml: 214; MCP-1 pg/ml: 21,7; MDC pg/ml: 14,6; MIP-1 beta pg/ml: 34,1; SCF pg/ml: 65,5; TNF-beta pg/ml: 6,55; TPO pg/ml: 1,050. For a list of all assessed markers (see Supplementary Table 2).

### CSF Measurement of Aβ_1−42_, Total-Tau, Phospho-Tau, NFL and Total α-Synuclein

CSF levels of Aβ_1−42_, *t-*Tau and p181-Tau were measured using ELISA kits from INNOTEST, Fujirebio GmbH, Germany. CSF levels of NFL were measured using the UmanDiagnostics NF-light^®^assay. Intra-assay coefficients of variation for each CSF parameter were below 15%. CSF levels of total α-synuclein were assessed using an ELISA kit for human α-synuclein (Roboscreen GmbH, Germany). Intra-assay imprecision of 4.4% was calculated from duplicate analyses and expressed as median of the range to average of the duplicates. Inter-assay imprecision of <10% was determined using two quality control CSF pool samples.

### Ethical Approval and Patient Consents

The study was approved by the Ethics Committee of the University of Tuebingen (26/2007BO1, 404/2010BO1, 199/2011BO1, 702/2013BO1, 428/2018BO2). All participants gave written informed consent.

### Data Availability

Anonymized data are available upon request to: kathrin.brockmann@uni-tuebingen.de.

### Statistical Analysis

Statistical analysis was performed using SPSS 26.0 software for Windows (IBM). All analyses were stratified by sex as the immune system shows relevant sex-specific differences of inflammatory profiles (23). Group comparisons (disease group vs. controls) of continuous data were analyzed using ANOVA/ANCOVA including age as co-variate where appropriate. Pearson's correlation was used to evaluate associations between inflammatory profiles in CSF and serum and between inflammatory profiles and CSF levels of α-synuclein, Aβ_1−42_, *t-*Tau, p181-Tau, and NFL. As this study was exploratory, we did not correct for multiple testing. However, only correlations with at least 10 valid sample pairs and a correlation coefficient ρ > 0.20 were considered meaningful (irrespective if the *p*-values was <0.05).

### Exclusion of Inflammatory Markers From Analyses

Of the 41 analyzed inflammatory markers some were not detectable in CSF and/or Serum. Specifically, Interleukin-5 and Interleukin-12p70 weren't detectable in CSF and Serum in healthy controls while Interleukin-12p70 wasn't detectable in CSF in PD patients. BDNF wasn't detectable in CSF in any of the cohorts. Therefore, these 3 metabolites were excluded from the respective group analyses.

Moreover, some inflammatory markers were measurable only in a small number (<10) of CSF/Serum pairs and/or of inflammatory/PD-biomarker pairs. These are listed below and were also excluded from the respected analyses due to lack of meaningful outcome.

#### Healthy Controls

For CSF/serum correlations of inflammatory markers, the following metabolites were excluded as the number of measurable CSF/serum pairs were <10:

Males: FactorVII (*n* = 7), TNF-alpha (*n* = 5), Interleukin-1 alpha (*n* = 0), Interleukin-1 beta (*n* = 0), Interleukin-2 (*n* = 0), Interleukin-3 (*n* = 0), Interleukin-7 (*n* = 1), Interleukin-10 (*n* = 9), Interleukin-12p40 (*n* = 4), Interleukin-13 (*n* = 7), Interleukin-15 (*n* = 8), Lymphotactin (*n* = 2), MDC (*n* = 2), TPO (*n* = 9), Alpha-fetoprotein (*n* = 2), CEA (*n* = 3), CKMB (*n* = 3), CA-125 (*n* = 5) and IgE (*n* = 0).

Females: FactorVII (*n* = 6), Interleukin-1 alpha (*n* = 8), Interleukin-1 beta (*n* = 4), Interleukin-2 (*n* = 7), Interleukin-3 (*n* = 0), Interleukin-7 (*n* = 5), Lymphotactin (*n* = 4), MDC (*n* = 1), Alpha-fetoprotein (*n* = 1), CEA (*n* = 4), CKMB (*n* = 2), PSA-f (*n* = 8), CA-125 (*n* = 3) and IgE (*n* = 3).

For correlations between CSF inflammatory markers and CSF PD-biomarkers the following metabolites were excluded as the number of measurable pairs were <10:

Males: FactorVII (*n* = 7), MMP9 (*n* = 8), TNF-alpha (*n* = 4), Interleukin-1 alpha (*n* = 4), Interleukin-1 beta (*n* = 0), Interleukin-2 (*n* = 1), Interleukin-3 (*n* = 1), Interleukin-7 (*n* = 4), Interleukin-12p40 (*n* = 8), Lymphotactin (*n* = 2), MDC (*n* = 2), TPO (*n* = 9), Alpha-fetoprotein (*n* = 2), CEA (*n* = 3), CKMB (*n* = 3), CA-125 (*n* = 5), IgE (*n* = 0).

Females: FactorVII (*n* = 6), Interleukin-1 beta (*n* = 4), Interleukin-3 (*n* = 0), Lymphotactin (*n* = 6), MDC (*n* = 1), Alpha-fetoprotein (*n* = 1), CEA (*n* = 2), CKMB (*n* = 2), CA-125 (*n* = 3), IgE (*n* = 4).

For correlations between serum inflammatory markers and CSF PD-biomarkers the following metabolites were excluded as the number of measurable pairs were <10:

Male: Interleukin-1-alpha (*n* = 4), Interleukin-2 (*n* = 2), Interleukin-3 (*n* = 1), Interleukin-7 (*n* = 2), Interleukin-13 (*n* = 7).

Female: Interleukin-2 (*n* = 7), Interleukin-3 (*n* = 0), Interleukin-7 (*n* = 7).

#### Parkinson's Disease

For CSF/serum correlations of inflammatory markers, the following metabolites were excluded as the number of measurable CSF/serum pairs were <10:

Males: Interleukin-2 (*n* = 3), Interleukin-3 (*n* = 0), Interleukin-5 (*n* = 0).

Females: Interleukin-1-beta (*n* = 9), Interleukin-2 (*n* = 3), Interleukin-3 (*n* = 0), Interleukin-5 (*n* = 0), MDC (*n* = 6), PSA-f (*n* = 7) and IgE (*n* = 6).

For the correlation between CSF inflammatory markers with clinical and CSF PD-biomarkers the following metabolites were excluded as the number of measurable pairs were <10:

Males: Interleukin-3 (*n* = 3), Interleukin-5 (*n* = 2), MDC (*n* = 9).

Females: Interleukin-1-beta (*n* = 9), Interleukin-3 (*n* = 1), Interleukin-5 (*n* = 3), MDC (*n* = 6), IgE (*n* = 6).

For the correlation between serum inflammatory markers with clinical and CSF PD-biomarkers the following metabolites were excluded as the number of measurable pairs were <10:

Males: Interleukin-2 (*n* = 7), Interleukin-3 (*n* = 7).

Females: Interleukin-2 (*n* = 4), Interleukin-3 (*n* = 4), Interleukin-5 (*n* = 7), Interleukin-12p70 (*n* = 9).

## Results

### Correlation Between CSF and Serum Inflammatory Markers

#### Healthy Controls

In males, 18 out of 41 and in females 24 out of 41 inflammatory markers reached a sufficient number of analysable CSF/serum pairs (*n* ≥ 10) for meaningful correlation analysis. A significant correlation between CSF and Serum was found only in a small proportion of the analyzed markers. Interleukin-4 and Leptin were positively correlated between CSF and serum in males and in females (males: Interleukin-4 rho = 0.705 *p* = 0.007, Leptin rho = 0.767 *p* = 0.002; females: Interleukin-4 rho = 0.634 *p* ≤ 0.001, Leptin rho = 0.500 *p* = 0.009). In males, ICAM-1 (rho = 0.553 *p* = 0.032), MIP-1 beta (rho = 0.521 *p* = 0.047), PSA-f (rho = 0.738 *p* = 0.004) and TSH (rho = 0.567 *p* = 0.043) were positively correlated between CSF and serum. In females, Interleukin-6 (rho = 0.423 *p* = 0.039), MCP-1 (rho = 0.424 *p* = 0.019), FABP (rho = 0.389 *p* = 0.050) and Interleukin-12p40 (rho = 0.832 *p* ≤ 0.001) were positively correlated between CSF and serum.

#### Parkinson's Disease

**Figure 1:**
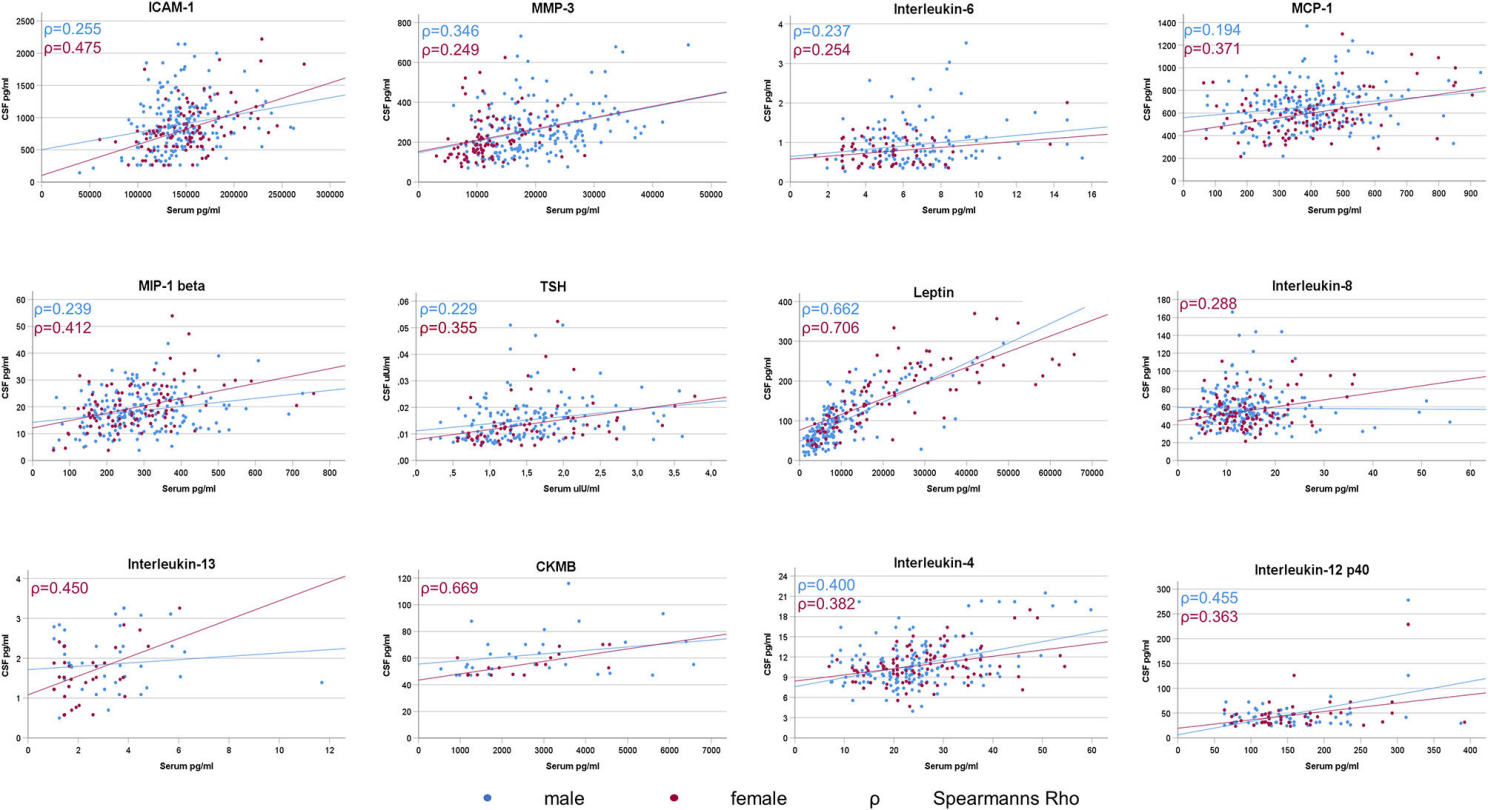
Significant CSF/Serum correlations of inflammatory markers in Parkinson's Disease. A significant correlation between CSF and serum levels was sparse and detected in only 25% (9 out of 36) of the analysable inflammatory markers in male PD patients and in only 38% (12 out of 32) of female PD patients.

In males, 36 out of 41 and in females 32 out of 41 markers reached a sufficient number of CSF/serum pairs (*n* ≥ 10) for meaningful correlation analysis. A positive correlation between CSF and Serum in male and in female PD patients was seen for ICAM-1 (males: rho = 0.255 *p* ≤ 0.001; females: rho = 0.475 *p* ≤ 0.001), MMP3 (males: rho = 0.346 *p* ≤ 0.001; females: rho = 0.249 *p* = 0.012), Interleukin-6 (males: rho = 0.237 *p* = 0.004; females: rho = 0.254 *p* = 0.030), MCP-1 (males: rho = 0.197 *p* = 0.007; females: rho = 0.371 *p* ≤ 0.001), MIP-1 beta (males: rho = 0.239 *p* ≤ 0.001; females: rho = 0.412 *p* ≤ 0.001), TSH (males: rho = 0.229 *p* = 0.004; females: rho = 0.355 *p* = 0.003), Leptin (males: rho = 0.662 *p* ≤ 0.001; females: rho = 0.706 *p* ≤ 0.001), Interleukin-4 (males: rho = 0.400 *p* ≤ 0.001; females: rho = 0.382 *p* ≤ 0.001) and Interleukin-12p40 (males: rho = 0.455 *p* ≤ 0.001; females: rho = 0.363 *p* = 0.006). In females also Interleukin-8 (rho = 0.288 *p* = 0.003), Interleukin-13 (rho = 0.450 *p* = 0.004) and CKMB (rho = 0.669 *p* = 0.003) were positively correlated (see Figure 1).

### Correlation Between CSF Inflammatory Markers With Demographics, Clinics and With PD-Biomarkers

#### Healthy Controls

There were no significant correlations between CSF inflammatory markers with age or with CSF levels of Aβ_1−42_, *t-*Tau, p181-Tau, NFL and α-synuclein in males and in females at the same time.

In males, higher CSF levels of ICAM-1, Interleukin-8 and SCF were associated with higher age. Higher CSF levels of ICAM-1, SCF, FABP, and TF were associated with higher CSF levels of *t-*Tau and p181-Tau. Low CSF levels of GH were associated with higher CSF levels *t-*Tau. Higher CSF levels of ICAM-1, Interleukin-8, SCF, and Leptin were associated with higher CSF levels of NFL. All correlation coefficients of these significant associations were between 0.5 and 0.9. There were no significant correlations between any of the inflammatory CSF markers with CSF levels of Aβ_1−42_ or α-synuclein in males.

In females, higher CSF levels of Leptin and lower CSF levels of ENA-78 were associated with higher age. Higher CSF levels of MMP3 and Interleukin-1 alpha were associated with higher CSF levels of Aβ_1−42_. Higher CSF levels of SCF and TF were associated with higher CSF levels α-synuclein. All correlation coefficients of these significant associations were between 0.4 and 0.7. There were no significant correlations between any of the inflammatory CSF markers with CSF levels of *t-*Tau, p181-Tau and NFL in females (see Table 1).

**Table 1:** Correlation between CSF inflammatory markers with demographics and CSF PD-biomarkers in healthy controls.

	**Age**	**Aβ_1−42_**	**t-Tau**	**p181-Tau**	**NFL**	**α-synuclein**
ENA-78	f: −0.463^*^	n.s.	n.s.	n.s.	n.s.	n.s.
FABP	n.s.	n.s.	m: 0.617^*^	m: 0.829^***^	n.s.	n.s.
GH	n.s.	n.s.	m: −0.659^*^	n.s.	n.s.	n.s.
ICAM-1	m: 0.524^*^	n.s.	m: 0.681^**^	m: 0.554^*^	m: 0.886^***^	n.s.
IL-1 alpha	n.s.	f: 0.686^*^	n.s.	n.s.	n.s.	n.s.
IL-8	m: 0.580^*^	n.s.	n.s.	n.s.	m: 0.696^*^	n.s.
Leptin	f: 0.392^*^	n.s.	n.s.	n.s.	m: 0.805^**^	n.s.
MMP3	n.s.	f: 0.501^**^	n.s.	m: 0.633^**^	n.s.	n.s.
SCF	m: 0.562^*^	n.s.	m: 0.643^**^	m: 0.640^**^	m: 0.780^**^	f: 0.739^**^
TF	n.s.	n.s.	m: 0.667^**^	m: 0.750^***^	n.s.	f: 0.705^*^

*Pearson correlation*:

* *p < 0.05*;

** *p < 0.01*;

*** *p ≤ 0.001*.

*m, males; f, females*.

*n.s., not significant*.

#### Parkinson's Disease

In both, males and females, higher CSF levels of ICAM-1, MMP3, SCF and FABP were associated with higher age. Moreover, higher CSF levels of GH were associated higher UPDRS-III scores and higher CSF levels of Interleukin-8 were associated with lower MoCA scores.

In males, higher CSF levels of CA-125 were associated with higher H&Y staging. Higher CSF levels of FABP were associated with lower MoCA scores.

In females, higher CSF levels of Interleukin-8 and lower CSF levels of Interleukin-18 were associated higher UPDRS-III scores and with H&Y staging. Higher CSF levels of MCP-1 and SCF were associated with lower MoCA scores.

All correlation coefficients of these significant associations were between 0.2 and 0.5 (see Table 2).

**Table 2:** Correlation between CSF inflammatory markers with demographics and clinical characteristics in Parkinson's Disease.

	**Age**	**Age at onset**	**Disease duration**	**H&Y**	**UPDRS-III**	**MoCA**	**LEDD**
CA-125	n.s.	n.s.	n.s.	m: 0.384^*^	n.s.	n.s.	n.s.
CEA	m: 0.284^*^	m: 0.332^**^	n.s.	n.s.	n.s.	n.s.	f: −0.478^*^
FABP	m: 0.539^***^ f: 0.304^**^	m: 0.511^***^ f: 0.236^*^	n.s.	n.s.	n.s.	m: −0.313^***^	n.s.
FactorVII	n.s.	n.s.	n.s.	n.s.	n.s.	n.s.	m: 0.396^**^
GH	n.s.	n.s.	n.s.	n.s.	m: 0.237* f: 0.301^*^	n.s.	n.s.
ICAM-1	m: 0.238^***^ f: 0.453^***^	f: 0.331^***^	f: 0.218^*^	n.s.	n.s.	n.s.	n.s.
IL-1 alpha	f: −0.316^*^	f: −0.387^*^	n.s.	n.s.	n.s.	n.s.	n.s.
IL-7	n.s.	n.s.	n.s.	n.s.	n.s.	n.s.	m: 0.259^**^
IL-8	f: 0.279^**^	n.s.	f: 0.406^***^	f: 0.312^***^	f: 0.267^**^	m: −0.232^***^ f: −0.255^**^	f: 0.262^**^
IL-18	n.s.	n.s.	n.s.	f: −0.348^**^	f: −0.452^***^		n.s.
MCP-1	f: 0.249^*^	m: 0.221^**^	n.s.	n.s.	n.s.	f: −0.294^**^	n.s.
MMP3	m: 0.294^***^ f: 0.276^**^	m: 0.268^***^ f: 0.304^**^	n.s.	n.s.	n.s.	n.s.	n.s.
PSA-f	m: 0.265^***^	m: 0.234^**^	n.s.	n.s.	n.s.	n.s.	m: 0.204^*^
SCF	m: 0.367^***^ f: 0.350^***^	m: 0.322^***^ f: 0.292^**^	n.s.	n.s.	n.s.	f: −0.272^**^	n.s.
TF	m: 0.403^***^	m: 0.389^***^	n.s.	n.s.	n.s.	n.s.	n.s.
TNF-alpha	f: −0.584^*^	f: −0.529^*^	n.s.	n.s.	n.s.	n.s.	f: 0.520^*^
TPO	n.s.	n.s.	f: 0.206^*^	n.s.	n.s.	n.s.	n.s.
TSH	n.s.	n.s.	n.s.	f: −0.266^*^	n.s.	n.s.	n.s.

*Pearson correlation*:

* *p < 0.05*;

** *p < 0.01*;

*** *p ≤ 0.001*.

*m, males; f, females*.

*n.s., not significant*.

In both, males and females, higher CSF levels of ICAM-1, MMP3, SCF, FABP, and TF were associated with higher CSF levels of *t-*Tau, p181-Tau, and α-synuclein. Moreover, higher CSF levels of FABP were associated with higher CSF levels of NFL.

All correlation coefficients of these significant associations were between 0.2 and 0.7 (see Table 3).

**Table 3:** Correlation between CSF inflammatory markers with CSF PD-biomarkers in Parkinson's Disease.

	**Aβ_1−42_**	**t-Tau**	**p181-Tau**	**NFL**	**α-synuclein**
CA-125	f: 0.614^*^	f: 0.595^*^	f: 0.637^*^	n.s.	n.s.
CEA	n.s.	n.s.	n.s.	m: 0.252^*^	n.s.
ENA-78	n.s.	n.s.	n.s.	n.s.	m: 0.225^**^ f: 0.276^*^
FABP	m: 0.215^**^	m: 0.701^***^ f: 0.486^***^	m: 0.555^***^ f: 0.425^***^	m: 0.300^***^ f: 0.209^*^	m: 0.453^***^ f: 0.426^***^
FactorVII	n.s.	f: 0.398^*^	f: 0.440^*^	n.s.	n.s.
GH	n.s.	n.s.	f: −0.298^*^	n.s.	n.s.
ICAM-1	n.s.	m: 0.347^***^ f: 0.543^***^	m: 0.301^***^ f: 0.525^***^	f: 0.219^*^	m: 0.360^***^ f: 0.521^***^
IL-1 alpha	n.s.	f: −0.437^**^	f: −0.372^*^	n.s.	f: −0.353^*^
IL-6	n.s.	n.s.	f: 0.259^*^	n.s.	n.s.
IL-8	n.s.	n.s.	n.s.	f: 0.364^***^	n.s.
IL-12p40	f: 0.370^**^	n.s.	n.s.	n.s.	n.s.
IL-16	n.s.	m: 0.200^**^	m: 0.228^**^	n.s.	m: 0.241^***^
Lymphotactin	f: 0.377^*^	n.s.	n.s.	n.s.	n.s.
MDC	m: 0.641^*^	m: 0.669^*^	m: 0.808^**^	n.s.	n.s.
MIP-1 beta	n.s.	f: 0.216^*^	n.s.	n.s.	n.s.
MMP3	m: 0.277^***^	m: 0.437^***^ f: 0.496^***^	m: 0.421^***^ f: 0.598^***^	n.s.	m: 0.384^***^ f: 0.490^***^
SCF	m: 0.227^***^	m: 0.625^***^ f: 0.651^***^	m: 0.573^***^ f: 0.643^***^	m: 0.264^***^	m: 0.542^***^ f: 0.589^***^
TF	m: 0.304^***^ f: 0.334^***^	m: 0.759^***^ f: 0.578^***^	m: 0.689^***^ f: 0.698^***^	m: 0.252^***^	m: 0.623^***^ f: 0.642^***^
TNF-alpha	n.s.	n.s.	n.s.	m: 0.338^*^	n.s.

*Pearson correlation*:

* *p < 0.05*;

** *p < 0.01*;

*** *p ≤ 0.001*.

*m, males; f, females*.

*n.s., not significant*.

### Correlation Between Serum Inflammatory Markers With Demographics, Clinics and With PD-Biomarkers

#### Healthy Controls

There were no correlations between serum inflammatory markers with age, Aβ_1−42_, *t-*Tau, p181-Tau, NFL or with α-synuclein in males and in females at the same time.

In males, higher serum levels of Interleukin-15 were associated with higher age. Lower serum levels of MMP3 were associated with higher CSF levels of NFL.

In females, higher serum levels of TNF-alpha, Interleukin-6, Interleukin-16, CEA and TF were associated with lower CSF levels of Aβ_1−42_. Higher serum levels of Interleukin-6, Interleukin-18, CEA and CA-125 were associated with lower CSF levels of p181-Tau. Higher serum levels of Factor VII, FABP, Leptin and TF were associated with higher CSF levels of NFL.

All correlation coefficients of these significant associations were between 0.3 and 0.9 (see Table 4).

**Table 4:** Correlation between serum inflammatory markers with demographics and CSF PD-biomarkers in healthy controls.

	**Age**	**Aβ_1−42_**	***t*-Tau**	**p181-Tau**	**NFL**	**α-synuclein**
CA-125	n.s.	f: −0.437^*^	n.s.	f: −0.381^*^	n.s.	n.s.
CEA	n.s.	f: −0.625^***^	n.s.	f: −0.433^*^	n.s.	n.s.
ENA-78	n.s.	f: 0.502^**^	n.s.	n.s.	n.s.	n.s.
FABP	n.s.	n.s.	n.s.	n.s.	f: 0.615^*^	n.s.
FactorVII	n.s.	n.s.	n.s.	n.s.	f: 0.655^*^	n.s.
IL-6	n.s.	f: −0.448^*^	n.s.	f: −0.394^*^	n.s.	n.s.
IL-15	m: −0.719^*^	n.s.	n.s.	n.s.	n.s.	n.s.
IL-16	n.s.	f: −0.407^*^	n.s.	n.s.	n.s.	n.s.
IL-18	n.s.	n.s.	f: −0.407^*^	f: −0.426^*^	n.s.	n.s.
Leptin	n.s.	n.s.	n.s.	n.s.	f: 0.735^*^	n.s.
MIP-1 beta	n.s.	n.s.	n.s.	n.s.	n.s.	f: −0.659^*^
MMP3	n.s.	n.s.	n.s.	n.s.	m: −0.735^*^	n.s.
TF	n.s.	f: −0.495^**^	n.s.	n.s.	f: 0.881^***^	n.s.
TNF-alpha	n.s.	f: −0.401^*^	n.s.	n.s.	n.s.	n.s.

*Pearson correlation*:

* *p < 0.05*;

** *p < 0.01*;

*** *p ≤ 0.001*.

*m, males; f, females*.

*n.s., not significant*.

#### Parkinson's Disease

In both, males and females, higher serum levels of FABP were associated with higher age whereas higher serum levels of CA-125 were associated with lower MoCA scores. No other correlation was found to be significant in both sexes at the same time.

In males, higher serum levels of Interleukin-13 and ENA-78 were associated with higher H&Y staging.

In females, higher serum levels of MMP3, TNF-alpha, Interleukin-15, FABP, CA-125 and lower serum levels of BDNF were associated with higher H&Y staging. Higher serum levels of TNF-alpha and Interleukin-15 were also associated with higher UPDRS-III scores. Moreover, higher serum levels of TNF-alpha and lower serum levels of BDNF and TSH were associated with lower MoCA scores.

All correlation coefficients of these significant associations were between 0.2 and 0.3 (see Table 5).

**Table 5:** Correlation between serum inflammatory markers with demographics and clinical characteristics in Parkinson's Disease.

	**Age**	**Age at onset**	**Disease duration**	**H&Y**	**UPDRS-III**	**MoCA**	**LEDD**
AFP	n.s.	m: 0.221^**^	n.s.		n.s.	n.s.	n.s.
BDNF	n.s.	n.s.	n.s.	f: −0.233^**^	n.s.	f: 0.214^**^	n.s.
CA-125	m: 0.214^**^	n.s.	n.s.	f: 0.223^*^	n.s.	m: −0.206^**^ f: −0.204^*^	n.s.
CEA	f: 0.248^*^	n.s.	f: 0.265^**^	n.s.	n.s.	n.s.	n.s.
CKMB	n.s.	n.s.	n.s.	n.s.	n.s.	n.s.	f: 0.310^**^
ENA-78	n.s.	n.s.	n.s.	m: 0.217^***^	n.s.	n.s.	n.s.
FABP	m: 0.317^***^ f: 0.343^***^	m: 0.255^***^	f: 0.352^***^	f: 0.269^**^	n.s.	m: −0.205^**^	n.s.
ICAM-1	f: 0.226^*^	n.s.	f: 0.206^*^	n.s.	n.s.	n.s.	n.s.
IL-1 alpha	n.s.	f: −0.307^**^	f: 0.311^**^	n.s.	n.s.	n.s.	n.s.
IL-3	n.s.	n.s.	f: 0.426^*^	n.s.	n.s.	n.s.	n.s.
IL-6	n.s.	n.s.	f: 0.348^***^	n.s.	n.s.	n.s.	n.s.
IL-7	n.s.	n.s.	f: 0.342^**^	n.s.	n.s.	n.s.	f: 0.306^*^
IL-8	f: 0.296^***^	f: 0.250^**^	n.s.	n.s.	n.s.	n.s.	n.s.
IL-12p40	n.s.	n.s.	n.s.	n.s.	n.s.	n.s.	f: 0.272^***^
IL-13	n.s.	n.s.	m: 0.289^***^	m: 0.239^**^	n.s.	n.s.	m: 0.201^*^
IL-15	n.s.	n.s.	f: 0.249^**^	f: 0.337^***^	f: 0.233^*^	n.s.	n.s.
IL-18	n.s.	n.s.	f: 0.205^*^	n.s.	n.s.	n.s.	n.s.
MMP3	f: 0.362^***^	f: 0.238^*^	f: 0.217^*^	f: 0.295^**^	n.s.	n.s.	n.s.
SCF	m: 0.305^***^	m: 0.255^***^	n.s.	n.s.	n.s.	n.s.	n.s.
TNF-alpha	n.s.	n.s.	n.s.	f: 0.203^*^	f: 0.206^*^	f: −0.254^**^	n.s.
TSH	n.s.	n.s.	n.s.	n.s.	n.s.	f: 0.205^*^	f: 0.233^*^

*Pearson correlation*:

* *p < 0.05*;

** *p < 0.01*;

*** *p ≤ 0.001*.

*m, males; f, females*.

*n.s., not significant*.

In both, males and females, higher serum levels of Interleukin-13 were associated with higher CSF levels of α-synuclein. There were no other significant correlations between serum inflammatory markers with Aβ_1−42_, *t-*Tau, p181-Tau, NFL or α-synuclein in males and in females at the same time.

In males, higher serum levels of Interleukin-7 were associated with higher CSF levels of p181-Tau and higher serum levels of Interleukin-10 were associated with higher CSF levels of α-synuclein.

In females, higher serum levels of Interleukin-6, Interleukin-15 and CKMB were associated with higher CSF levels Aβ_1−42_. Higher serum levels of ICAM-1, Interleukin-18, and MIP-1 beta were associated with higher CSF levels of *t-*Tau and p181-Tau. Higher serum levels of MMP3, TNF-alpha, Interleukin-6, FABP, TF, CA-125, and IgE were associated with higher CSF levels of NFL. Higher serum levels of ICAM-1, Interleukin-12p40, Interleukin-16, Interleukin-18, MCP-1, and MIP-1 beta were associated with higher CSF levels of α-synuclein.

All correlation coefficients of these significant associations were between 0.2 and 0.4 (see Table 6).

**Table 6:** Correlation between serum inflammatory markers with CSF PD-biomarkers in Parkinson's Disease.

	**Aβ_1−42_**	***t*-Tau**	**p181-Tau**	**NFL**	**α-synuclein**
CA-125	n.s.	n.s.	n.s.	f: 0.275^**^	n.s.
CKMB	f: 0.297^**^	n.s.	n.s.	n.s.	n.s.
FABP	n.s.	n.s.	n.s.	f: 0.356^***^	n.s.
ICAM-1	n.s.	f: 0.254^**^	f: 0.213^*^	n.s.	f: 0.328^***^
IgE	n.s.	n.s.	n.s.	f: 0.244^*^	n.s.
IL-6	f: 0.220^*^	n.s.	n.s.	f: 0.219^*^	n.s.
IL-7	n.s.	n.s.	m: 0.266^*^	n.s.	n.s.
IL-10	n.s.	n.s.	n.s.	n.s.	m: 0.222^**^
IL-12p40	n.s.	n.s.	n.s.	n.s.	f: 0.273^**^
IL-13	n.s.	n.s.	n.s.	n.s.	m: 0.393^***^ f: 0.327^*^
IL-15	f: 0.247^*^	n.s.	n.s.	n.s.	n.s.
IL-16	n.s.	n.s.	n.s.	n.s.	f: 0.211^*^
IL-18	n.s.	f: 0.203^*^	f: 0.203^*^	n.s.	f: 0.214^*^
MCP-1	n.s.	n.s.	n.s.	n.s.	f: 0.246^*^
MIP-1 beta	n.s.	f: 0.317^***^	f: 0.287^**^	n.s.	f: 0.265^**^
MMP3	n.s.	n.s.	n.s.	f: 0.283^**^	n.s.
TF	n.s.	n.s.	n.s.	f: 0.268^**^	n.s.
TNF-alpha	n.s.	n.s.	n.s.	f: 0.264^**^	n.s.
TPO	n.s.	n.s.	n.s.	f: −0.294^**^	n.s.

*Pearson correlation*:

* *p < 0.05*;

** *p < 0.01*;

*** *p ≤ 0.001*.

*m, males; f, females*.

*n.s., not significant*.

## Discussion

By using a multiplex assay and assessing 41 inflammatory markers in CSF/serum pairs in 453 sporadic PD patients we show that:

- A significant correlation between CSF and serum was sparse and detected in only 25% (9 out of 36) of the analysable inflammatory markers in male PD patients and in only 38% (12 out of 32) of female PD patients. Of these markers, ICAM-1, Interleukin-4, Interleukin-6, Interleukin-12p40, MCP-1, MIP-1 beta, MMP3, Leptin, and TSH were associated in CSF and serum in both sexes whereas Interleukin-8, Interleukin-13 and CKMB were additionally correlated only in females.
- Higher CSF levels of FABP, Interleukin-8, MCP-1, and SCF were associated with older age and with cognitive impairment as measured by MoCA scores in males and/or females. Higher CSF levels of Interleukin-8 were also associated with motor impairment in UPDRS-III and H&Y staging.
- Higher CSF levels of FABP, ICAM-1, MMP3, SCF, and TF were associated with higher CSF levels of neurodegenerative/PD-specific biomarkers, namely *t-*Tau, p181-Tau, and α-synuclein in males and in females.
- Higher serum levels of CA-125, FABP, and TNF-alpha as well as lower serum levels of BDNF were associated with cognitive impairment as measured by MoCA scores and with motor impairment assessed by H&Y staging in males and/or females.
- Whereas some inflammatory serum markers were positively associated with CSF levels of *t-*Tau and p181-Tau (ICAM-1, Interleukin-18, MIP-1 beta) others were correlated with NFL (MMP3, TNF-alpha, FABP, TF, CA-125). Notably, all of these were only found in female PD patients and were not the same as those associated with the PD-specific biomarker CSF α-synuclein (Interleukin-10, Interleukin-12p40, Interleukin-13, Interleukin-16, Interleukin-18, MCP-1, MIP-1 beta).

Overall, associations between inflammatory markers with clinical outcomes and with CSF levels of neurodegenerative/PD-biomarkers were stronger and more robust for CSF than for serum.

It has already been shown in sporadic and in LRRK2-PD patients that higher serum levels of IL-8, IL-10, MCP-1, MIP-1, TNF-alpha are associated with more severe motor impairment assessed with UPDRS-III, Timed-up and Go and H&Y staging (3, 24, 25). Moreover, higher IL-8 plasma levels were associated with dementia in PD patients carrying a homo- or heterozygous mutation in the glucocerebrosidase (*GBA*) gene (PD_GBA_) (26). Here, we further support the role of these inflammatory markers to be associated with clinical outcomes of motor and cognitive performance and to be associated with CSF levels of neurodegenerative/PD-specific biomarkers. We could previously also demonstrate a role of FABP in sporadic as well as in LRRK2-associated PD with increased serum levels compared to healthy controls. This indicates a common disease-specific pattern irrespective of the underlying genotype (23). Now, we extend these findings and show that higher levels of FABP in CSF and in serum are also associated with clinical characteristics of motor and cognitive decline as well as with CSF levels of neurodegenerative biomarkers.

FABP is primarily involved in the intracellular transport of long-chain fatty acids (27). It was previously shown that α-synuclein binds to long-chain fatty acids resulting in enhanced α-synuclein oligomerization and Lewy body formation in dopaminergic neurons. FABP overexpression aggravated fatty acid-induced α-synuclein oligomerization in a mouse model (28). Although increased serum and CSF levels of FABP are found in Lewy body diseases, similar results were also observed in patients with stroke, brain injury and Creutzfeldt-Jakob disease, suggesting that FABP is a disease-non-specific pro-inflammatory marker (29–31). *In-vivo* and *in-vitro* experiments show an up-regulation of the Stem Cell Factor SCF in neurons of injured brain tissue paralleled by neural stem/progenitor cell migration highlighting that SCF is involved in self-renewal and cell survival (32). Apart from FABP and SCF, the other pro-inflammatory mediators found to be most important in our study (ICAM-1, IL-8, MCP-1, MIP-1-beta) represent pro-inflammatory chemokines of the central-nervous (microglia) and peripheral innate immune system (monocyte-macrophage-lineage). The intercellular adhesion molecule ICAM-1 has been demonstrated already several years ago in sustaining neuroinflammation via activated microglia in PD brains, MPTP-treated monkeys and rats (33, 34). IL-8 is produced by macrophages and promotes chemotaxis causing granulocytes to migrate toward sites of infectious/injured tissue where, as a second function of IL-8, phagocytosis is induced. Interestingly, secretion of IL-8 is increased by oxidative stress which promotes inflammation and thereby further increases oxidative stress. MIP-1 is also produced by macrophages and promotes chemotaxis and synthesis of other pro-inflammatory cytokines such as IL-1, IL-6 and TNF-alpha (35) whereas MCP-1 has a chemotactic function on monocytes.

So far, there is only one small study published assessing a variety of inflammatory markers by multiplex assay in CSF/serum pairs with further analyses in relation to clinical outcomes and CSF levels of neurodegenerative/PD-biomarkers (12). This sporadic PD cohort comprised 22 patients with paired CSF/serum sampling and was similar in age (mean 65.4 years) and disease duration (mean 5.4 years) to our cohort. Also, the assessed inflammatory markers (IFN-γ, IL-1β, IL-2, IL-4, IL-6, IL-8, IL-10, IL-12p70, IL-13, TNF-α) and the neurodegenerative/PD-biomarkers in CSF (Aβ_1−42_, *t-*Tau, pThr231-Tau, total α-synuclein) showed a huge overlap with the markers assessed in our study so that these 2 studies are nicely comparable. Importantly, results in our large PD cohort support and expand findings from that study: (I) Only a fraction of markers is robustly detectable in both, CSF and serum. (II) Correlations of cytokines between CSF and blood are sparse indicating that levels of central-nervous and peripheral inflammatory markers might be regulated independently of each other and that changes may not simply reflect passive diffusion of circulating cytokines into or out of the CNS. (III) Associations between inflammatory markers with CSF levels of neurodegenerative/PD-biomarkers are primarily seen with *t-*Tau, p-Tau and α-synuclein but not with Aβ_1−42_ and are more robust for CSF than for serum. These findings suggest that CSF levels of inflammatory markers better reflect central-nervous-system pathology as compared to peripheral inflammatory markers.

Based on results from the present study, further analyses are needed in order to design clinical trials aiming at disease-modification by targeting the immune system. Such studies should be longitudinal and address the following questions: Do blood/CSF inflammatory profiles change over the course of the disease and do they mirror the degree of neuroinflammation, respectively? Which blood/CSF inflammatory profiles are best associated with longitudinal phenotypical trajectories and what is their prognostic value? Which inflammatory markers in blood/CSF are most meaningful with regard to patient stratification and outcome measures? These might not necessarily be the same and it might not be a single marker but rather a combination. Promising candidates identified in the present study are FABP, ICAM-1, IL-8, MCP-1, MIP-1-beta, and SCF.

The strength of the present study is the large monocentric collection of high-quality CSF/serum pair samples according to standardized procedures which minimizes variance in sample collection and processing.

Limitations of our study are: (I) The single measurement of inflammatory markers so that variations in measurement cannot be accounted. (II) The mean storage-time until measurement of inflammatory markers was 6 years which might impact detectability of markers that are present at low concentrations.

## Data Availability Statement

Anonymized data are available upon request to: kathrin.brockmann@uni-tuebingen.de.

## Ethics Statement

The studies involving human participants were reviewed and approved by the Ethics Committee of the University of Tuebingen (26/2007BO1, 404/2010BO1, 199/2011BO1, 702/2013BO1, and 428/2018BO2). The patients/participants provided their written informed consent to participate in this study.

## Author Contributions

SL: conception, statistical analysis, manuscript preparation, and critical review of the manuscript. MZ, IW, BR, FF, and TG: execution of the study (clinical and lumbar puncture) and critical review of the manuscript. CD: execution of the study and critical review of the manuscript. KW and IL: execution of the study (hSYN ELISA) and critical review of the manuscript. MJ, TJ, and NS-M: analysis of inflammatory markers and critical review of the manuscript. KB: conception, design and execution of the study (clinical and lumbar puncture), statistical analysis, and manuscript preparation. All authors contributed to the article and approved the submitted version.

## Funding

This work was funded by the Michael J. Fox Foundation for Parkinson's Research (MJFF) within the grant *Influence of Inflammatory profiles on PD Phenotype and Progression*. Moreover, it was partially funded by the PD-Strat project (FKZ 031L0137B) which was supported by the German Federal Ministry of Education and Research (BMBF) in the frame of ERACoSysMed2.

## Conflict of Interest

KW and IL are employed by Roboscreen GmbH which manufactures the ELISA kit for measurements of total human α-synuclein used in the present study. The remaining authors declare that the research was conducted in the absence of any commercial or financial relationships that could be construed as a potential conflict of interest.

## Publisher's Note

All claims expressed in this article are solely those of the authors and do not necessarily represent those of their affiliated organizations, or those of the publisher, the editors and the reviewers. Any product that may be evaluated in this article, or claim that may be made by its manufacturer, is not guaranteed or endorsed by the publisher.
