# Supplementary material for: CSF and Serum Levels of Inflammatory Markers in PD: Sparse Correlation, Sex Differences and Association With Neurodegenerative Biomarkers
Source: Front Neurol. 2022 Feb 25;13:834580. doi: 10.3389/fneur.2022.834580 (PMC8914943; doi:10.3389/fneur.2022.834580)
Supplement: Supplementary file 1 [file Table_1.docx]

**Supplemental Table 1: Demographics and clinical data**

|  | Healthy controls | Parkinson`s Disease | p-value |
| --- | --- | --- | --- |
| **males** | | | |
|  | N=15 | N=298 |  |
| Age | 65 ± 12 | 66 ± 9 | 0.822 |
| Age at onset | n.a. | 59 ± 10 | n.a. |
| Disease duration | n.a. | 7 ± 4 | n.a. |
| H&Y | n.a. | 2.2 0.6 | n.a. |
| UPDRS-III | n.a. | 28 ± 12 | n.a. |
| MoCA | n.a. | 25 ± 4 | n.a. |
| LEDD | n.a. | 619 ± 479 | n.a. |
| Aβ_1-42_ | 763 ± 306 | 676 ± 263 | 0.224 |
| t-Tau | 260 ± 89 | 230 ± 119 | 0.345 |
| p181-Tau | 47 ± 147 | 41 ± 18 | 0.194 |
| NFL | 761 ± 304 | 1025 ± 873 | 0.342 |
| α-synuclein | 592 ± 212 | 569 ±284 | 0.669 |
| **females** | | | |
|  | N=33 | N=155 |  |
| Age | 55 ± 16 | 66 ± 9 | ≤0.001 |
| Age at onset | n.a. | 59 ± 10 | n.a. |
| Disease duration | n.a. | 7 ± 5 | n.a. |
| H&Y | n.a. | 2.1 ± 0.7 | n.a. |
| UPDRS-III | n.a. | 26 ± 12 | n.a. |
| MoCA | n.a. | 25 ± 5 | n.a. |
| LEDD | n.a. | 533 ± 374 | n.a. |
| Aβ_1-42_ | 602 ± 266 | 684 ± 238 | 0.106^a^ |
| t-Tau | 188 ± 104 | 257 ± 151 | 0.952^a^ |
| p181-Tau | 38 ± 19 | 43 ± 17 | 0.411^a^ |
| NFL | 588 ± 235 | 1143 ± 1920 | 0.757^a^ |
| α-synuclein | 718 ± 291 | 689 ± 377 | 0.141^a^ |

^a^ ANCOVA: age as covariate

n.a. = not applicable
