# Supplementary material for: CSF and Serum Levels of Inflammatory Markers in PD: Sparse Correlation, Sex Differences and Association With Neurodegenerative Biomarkers
Source: Front Neurol. 2022 Feb 25;13:834580. doi: 10.3389/fneur.2022.834580 (PMC8914943; doi:10.3389/fneur.2022.834580)
Supplement: Supplementary file 2 [file Table_2.docx]

**Supplemental Table 2: CSF and Serum Inflammatory marker included in the multiplex assay**

|  | Inflammatory marker | Abbreviation | n = measurable in CSF | n = measurable in Serum |
| --- | --- | --- | --- | --- |
| 1 | Alpha Fetoprotein | AFP | HC: 3; PD: 32 | HC: 45; PD: 296 |
| 2 | Brain Derived Neurotrophic Factor | BDNF | HC: 0; PD: 0 | HC: 48; PD: 453 |
| 3 | Cancer Antigen 125 | CA-125 | HC: 8; PD: 41 | HC: 45; PD: 303 |
| 4 | Carcinoembryonic Antigen | CEA | HC: 7; PD: 97 | HC: 46; PD: 300 |
| 5 | Creatine Kinase Muscle Brain type | CKMB | HC: 5; PD: 47 | HC: 46; PD: 300 |
| 6 | Epithelial-derived neutrophil-activating peptide 78 | ENA-78 | HC: 29; PD: 199 | HC: 47; PD: 451 |
| 7 | FactorVII | FactorVII | HC: 13; PD: 83 | HC: 46; PD: 303 |
| 8 | Fatty Acid Binding Protein | FABP | HC: 45; PD: 275 | HC: 44; PD: 297 |
| 9 | Growth Hormone | GH | HC: 27; PD: 152 | HC: 43; PD: 296 |
| 10 | Immunoglobulin E | IgE | HC: 4; PD: 18 | HC: 41; PD: 287 |
| 11 | Intercellular Adhesion Molecule 1 | ICAM-1 | HC: 46; PD: 299 | HC: 46; PD: 302 |
| 12 | Interleukin-1 alpha | IL-1 alpha | HC: 17; PD: 125 | HC: 18; PD: 229 |
| 13 | Interleukin-1 beta | IL-1 beta | HC: 4; PD: 45 | HC: 45; PD: 297 |
| 14 | Interleukin-2 | IL-2 | HC: 17; PD: 42 | HC: 9; PD: 12 |
| 15 | Interleukin-3 | IL-3 | HC: 1; PD: 3 | HC: 3; PD: 88 |
| 16 | Interleukin-4 | IL-4 | HC: 46; PD: 301 | HC: 46; PD: 437 |
| 17 | Interleukin-5 | IL-5 | HC: 0; PD: 5 | HC: 0; PD: 25 |
| 18 | Interleukin-6 | IL-6 | HC: 37; PD: 224 | HC: 46; PD: 298 |
| 19 | Interleukin-7 | IL-7 | HC: 21; PD: 167 | HC: 15; PD: 199 |
| 20 | Interleukin-8 | IL-8 | HC: 46; PD: 300 | HC: 46; PD: 444 |
| 21 | Interleukin-10 | IL-10 | HC: 27; PD: 214 | HC: 35; PD: 360 |
| 22 | Interleukin-12p40 | IL-12p40 | HC: 31; PD: 179 | HC: 38; PD: 393 |
| 23 | Interleukin-12p70 | IL-12p70 | HC: 0; PD: 0 | HC: 0; PD: 32 |
| 24 | Interleukin-13 | IL-13 | HC: 43; PD: 270 | HC: 21; PD: 224 |
| 25 | Interleukin-15 | IL-15 | HC: 40; PD: 247 | HC: 33; PD: 316 |
| 26 | Interleukin-16 | IL-16 | HC: 43; PD: 265 | HC: 48; PD: 450 |
| 27 | Interleukin-18 | IL-18 | HC: 30; PD: 174 | HC: 48; PD: 446 |
| 28 | Leptin | Leptin | HC: 41; PD: 282 | HC: 45; PD: 299 |
| 29 | Lymphotactin | Lymphotactin | HC: 10; PD: 96 | HC: 26; PD: 205 |
| 30 | Macrophage Derived Chemokine | MDC | HC: 3; PD: 16 | HC: 48; PD: 452 |
| 31 | Macrophage Inflammatory Protein 1 Beta | MIP-1 beta | HC: 45; PD: 292 | HC: 48; PD: 446 |
| 32 | Matrix Metallopeptidase 3 | MMP3 | HC: 46; PD: 298 | HC: 46; PD: 300 |
| 33 | Matrix Metallopeptidase 9 | MMP9 | HC: 26; PD: 101 | HC: 46; PD: 303 |
| 34 | Monocyte Chemoattractant Protein 1 | MCP-1 | HC: 46; PD: 297 | HC: 47; PD: 449 |
| 35 | Prostate Specific Antigen (free) | PSA-f | HC: 27; PD: 172 | HC: 29; PD: 196 |
| 36 | Stem Cell Factor | SCF | HC: 46; PD: 300 | HC: 48; PD: 452 |
| 37 | Thyreoperoxidase | TPO | HC: 36; PD: 265 | HC: 48; PD: 452 |
| 38 | Thyroid Stimulating Hormone | TSH | HC: 34; PD: 223 | HC: 46; PD: 297 |
| 39 | Tissue Factor | TF | HC: 45; PD: 298 | HC: 46; PD: 304 |
| 40 | Tumor Necrosis Factor alpha | TNF-alpha | HC: 15; PD: 52 | HC: 46; PD: 303 |
| 41 | Tumor Necrosis Factor beta | TNF-beta | HC: 33; PD: 222 | HC: 38; PD: 361 |
